# Editorial: Positron Emission Tomography (PET) Imaging of Brain Biochemistry: Beyond High-Affinity Radioligands

**DOI:** 10.3389/fnins.2022.907460

**Published:** 2022-05-03

**Authors:** Michael R. Kilbourn, Peter J. H. Scott, Neil Vasdev

**Affiliations:** ^1^Department of Radiology, University of Michigan, Ann Arbor, MI, United States; ^2^Azrieli Centre for Neuro-Radiochemistry, Brain Health Imaging Centre, Centre for Addiction and Mental Health and Department of Psychiatry, University of Toronto, Toronto, ON, Canada

**Keywords:** brain PET, neuroimaging, radiochemistry, radiopharmaceuticals, molecular imaging

The field of radiopharmaceutical chemistry devoted to the development of new neuroimaging agents for Positron Emission Tomography (PET) imaging began in earnest in the early 1980's and was for many years dominated by efforts targeting receptors for the major neurotransmitters such as biogenic amines, amino acids, and acetylcholine. As new synthetic methods were developed for radiolabeling with short-lived radionuclides such as carbon-11 and fluorine-18, attention was directed to the myriad of other possibilities for imaging important facets of physiology, such as enzymes and misfolded proteins. In this Research Topic, papers were selected to illustrate both the possibilities and the challenges of new PET radiotracer development, while also highlighting non-traditional imaging targets currently being investigated.

The contribution from Zeng et al. demonstrates many of the techniques used to evaluate new brain radiotracers targeting receptors, but with significant challenges not seen with most systems: the 5HT_2C_ receptor is but one of 14 subtypes of serotonin receptors and obtaining the encouraging subtype specificity achieved in this work has historically been difficult. The *in vivo* applications of such radiotracers are further complicated by the difficulties in imaging such target regions as the choroid plexus, a thin layer of cells in the brain.

PET imaging of misfolded proteins has garnered enormous attention in recent years, yielding radiopharmaceuticals for imaging amyloid and tau in neurodegenerative disorders such as Alzheimer's disease and related dementias. Several of these have gained U.S. Food and Drug Administration approval for clinical use, and additional radiotracers for “holy grail targets” such as α-synuclein have advanced to clinical trials. Building on these efforts, a number of research groups are developing radiotracers for additional misfolded proteins. The two contributions from Lindberg et al. and Kaur et al. exemplify the targeting of proteins in the brain that have nothing to do with classical neurotransmission, but which have been recognized as crucial aspects of neurological diseases.

Microtubules are an important component of cellular structures in the brain, and the loss of microtubule function is apparent in a variety of neurodegenerative diseases. The studies of [^11^C]verubulin reported here demonstrate the challenges of interpreting unexpected results when multiple species are used in the evaluation of new radiotracers.

As discussed by Kaur et al., the hallmark of Huntington's disease is the aggregation of a form of the huntingtin protein, produced by a mutant huntingtin gene. There are no “receptor” binding sites on such aggregated proteins, but in a manner analogous to the development of radiotracers for β-amyloid, tau, and α-synuclein, the authors have successfully identified a [^18^F]trifluoromethyl-labeled small molecule that shows saturable *in vitro* binding to huntingtin aggregates, and suitable permeability through the mammalian blood-brain-barrier.

Finally, there has also been considerable interest in imaging enzyme activity since the early days of brain PET, including seminal work quantifying hexokinase ([^18^F]fludeoxyglucose) and monoamine oxidase activity in a variety of neuropsychiatric disorders and addictions. The latter continues to garner much attention today, and the contribution from Meyer and Braga presents an up-to-date review of the successful development of a variety of radiotracers targeting the enzymes monoamine oxidase-A and -B.

This Research Topic of articles presents the multiple approaches to brain imaging with PET that are in use today. The papers illustrate how, after almost 50 years since the first human brain imaging studies with [^18^F]fludeoxyglucose, the lessons learned continue to be applied to the emerging targets implicated in neurological and neuropsychiatric disorders. The authors show that new developments in PET radiochemistry are increasing the chemical space that can be radiolabeled with positron-emitting radionuclides, offering access to novel radiotracers. However, the evaluation of the new tracers reported shows that, in many ways, the challenges of developing *in vivo* radiotracers for imaging new targets in the brain remain the same as they did 40 or 50 years ago: development of imaging agents with appropriate CNS penetration, target engagement, high affinity (B_max_/K_d_ > 5) and selectivity, suitable brain pharmacokinetics, and acceptable metabolism. Many of the tracers reported in this Research Topic of articles pave the way to explore brain biochemistry by imaging beyond typical high-affinity targets. We are excited to watch the progress of not only the new radiotracers reported here, but also the many future ones sure to be developed as the improving methods for PET radiochemistry and *in vivo* radiotracer evaluation continue to produce unprecedented radiotracers to be translated to *in vivo* studies in the human brain.

## Author Contributions

All authors contributed to this Editorial and approved the final version.

## Conflict of Interest

The authors declare that the research was conducted in the absence of any commercial or financial relationships that could be construed as a potential conflict of interest.

## Publisher's Note

All claims expressed in this article are solely those of the authors and do not necessarily represent those of their affiliated organizations, or those of the publisher, the editors and the reviewers. Any product that may be evaluated in this article, or claim that may be made by its manufacturer, is not guaranteed or endorsed by the publisher.

